# Human *mecC*-Carrying MRSA: Clinical Implications and Risk Factors

**DOI:** 10.3390/microorganisms8101615

**Published:** 2020-10-20

**Authors:** Carmen Lozano, Rosa Fernández-Fernández, Laura Ruiz-Ripa, Paula Gómez, Myriam Zarazaga, Carmen Torres

**Affiliations:** Area of Biochemistry and Molecular Biology, University of La Rioja, 26006 Logroño, Spain; rosa.fernandez.1995@gmail.com (R.F.-F.); laura_ruiz_10@hotmail.com (L.R.-R.); paula_gv83@hotmail.com (P.G.); myriam.zarazaga@unirioja.es (M.Z.); carmen.torres@unirioja.es (C.T.)

**Keywords:** *Staphylococcus aureus*, methicillin resistance, human infection, CC130

## Abstract

A new methicillin resistance gene, named *mecC*, was first described in 2011 in both humans and animals. Since then, this gene has been detected in different production and free-living animals and as an agent causing infections in some humans. The possible impact that these isolates can have in clinical settings remains unknown. The current available information about *mecC*-carrying methicillin resistant *S. aureus* (MRSA) isolates obtained from human samples was analyzed in order to establish its possible clinical implications as well as to determine the infection types associated with this resistance mechanism, the characteristics of these *mecC*-carrying isolates, their possible relation with animals and the presence of other risk factors. Until now, most human *mecC*-MRSA infections have been reported in Europe and *mecC*-MRSA isolates have been identified belonging to a small number of clonal complexes. Although the prevalence of *mecC*-MRSA human infections is very low and isolates usually contain few resistance (except for beta-lactams) and virulence genes, first isolates harboring important virulence genes or that are resistant to non-beta lactams have already been described. Moreover, severe and even fatal human infection cases have been detected. *mecC*-carrying MRSA should be taken into consideration in hospital, veterinary and food safety laboratories and in prevention strategies in order to avoid possible emerging health problems.

## 1. Introduction

*Staphylococcus aureus* is an opportunistic pathogen that causes high morbidity and mortality. This microorganism is able to cause diverse diseases that range from having a relatively minor impact, such as a skin infection, to serious and life-threatening episodes, such as endocarditis, pneumonia or sepsis. The impact of *S. aureus* is enhanced by its great capacity to develop and acquire resistance to various antimicrobial agents. Among the antibiotic resistance of *S. aureus*, methicillin resistance mediated by the *mecA* gene is highly relevant as this mechanism provides this bacterium resistance to almost all beta-lactam antibiotics, seriously limiting therapeutic options [1,2]. Recently, the World Health Organization (WHO) outlined the greatest threats in terms of antimicrobial resistance and methicillin-resistant *S. aureus* (MRSA) was classified as a high-priority microorganism. For many years, MRSA infections were only reported in hospitals, with it being considered to be a nosocomial pathogen (hospital-associated MRSA or HA-MRSA). In the 1990s, community-associated MRSA (CA-MRSA) cases in healthy humans without any connection to healthcare settings started to be described and, nowadays, the distinction between CA-MRSA and HA-MRSA seems to be disappearing [3,4]. 

For the last two decades, a third epidemiological group known as livestock-associated MRSA (LA-MRSA) has been described. *S. aureus* has been considered to be an important zoonotic agent with a great capacity to cause infections in different animal species and in humans. Various studies have suggested that there is a high specificity of the different genetic lineages of *S. aureus* for the host [5]. However, many cases of clones related to animals have been detected and have caused infections in humans [6,7]. Presently, different clonal lineages associated with LA-MRSA have been described and, among these, the clonal complex (CC) CC398 stands out (Table S1). CC398 is related to production animals, mainly pigs, and has been detected worldwide [8]. Infection cases have been identified in humans, both in contact and without contact with animals [9,10,11]. In addition to CC398, there are other clonal complexes associated with animals such as CC5 in birds, CC9 in pigs, CC97 in cattle or CC133 in small ruminants [12,13,14,15]. 

Remarkably, a new methicillin resistance gene (*mecA*_LGA251_, which shares only 70% similarity to *mecA* (Figure S1)) was first described in 2011 in both humans and animals [16,17]. Initially these strains were associated with dairy cows and these animals were considered to be a possible reservoir [16]. Since then, this gene has been detected in different production and free-living animals and as an agent causing infection in some humans [8,18]. This new gene was named *mecC* since *mecB* had previously been described in macrococci, but not in staphylococcal species [19]. Worryingly, *mecB* has been recently identified in *S. aureus* and future studies should determine the potential risk that this entails [20]. In the case of MRSA isolates carrying the *mecC* gene (*mecC*-MRSA isolates), these isolates have already been identified as belonging to diverse clonal lineages such as CC130, CC49, sequence type (ST) 151, ST425, CC599 or CC1943 and in very different hosts, including its detection in environmental samples [8,21,22,23]. There are different theories about the origin of the *mecC* gene and the possible impact that these isolates can have in clinical settings. In this review, the objective was to describe current knowledge about *mecC* detection in humans and its possible clinical implications, as well as to determine the infection types associated with this resistance mechanism, the characteristics of these *mecC*-carrying isolates, their possible relationship with animals and the presence of other risk factors.

## 2. Detection of *mecC*-MRSA Isolates in Humans

### 2.1. Human Studies Related to mecC-MRSA

Although the *mecC* gene was initially discovered in an isolate from bulk milk in England, the first human *mecC*-MRSA isolates were also identified in that same study [16]. These human isolates were obtained from patients from the United Kingdom and Denmark. Moreover, in a publication from the same year, two human MRSA isolates carrying this new resistance gene were independently identified in Ireland [17].

Since then, several retrospective and prospective studies using human *S. aureus* isolates/samples were carried out in order to search for *mecC*-MRSA isolates (Table 1 and Table 2) [16,18,24,25,26,27,28,29,30,31,32,33,34,35,36,37,38,39,40,41,42,43,44,45,46,47,48,49,50,51,52,53,54,55,56,57,58,59,60,61,62,63,64,65,66,67,68,69,70,71,72,73,74]. Most of these studies were performed in European countries (Table 1 and Table 2), and the UK and Denmark were the countries in which the highest levels of *mecC*-MRSA isolates were detected [16,24,25,39,41].

Unfortunately, the design of these studies was very different, which complicates any comparison of the data obtained. Importantly, the criteria chosen for the selection of the initial isolates/samples varied significantly. While all *S. aureus* isolates were collected in some studies [29,38,45], only MRSA isolates were included in others [24,26,27,28,32,33,36,41,42]. Moreover, several studies were more restrictive and only used isolates that showed characteristics suspected of carrying the *mecC* gene such as *spa* types associated with *mecC*-positive clonal lineages previously described, *mecA*-negative MRSA isolates, isolates with antimicrobial susceptibility suspected to be *mecC*-positive or *pvl*-negative MRSA isolates [25,26,37,69] (Table S1). In any case, the *mecC*-MRSA human prevalence detected in most of the studies was very low. Several studies did not identify any *mecC*-positive *S. aureus* among included human isolates/samples (Table 2) [45,46,47,48,49,50,51,52,53,54,55,56,57,58,59,60,61,62,63,64,65,66,67,68,69,70,71,72,73,74]. In studies in which this gene was detected (Table 1), the prevalence identified, considering the total number of isolates/samples included, was < 1% in most of the cases [24,27,28,29,32,33,37,40,41,42,43], similar to that identified in the first study in which *mecC* was discovered (approximately 0.04%) [16]. In a few studies, the prevalence was > 1% but, in all of these, only a small number of initial isolates (<400 isolates) was used; this may be the reason for the high prevalence value obtained (up to 6.3%) [25,26,36,38,39]. Recently, a meta-analysis of the prevalence of *mecC*-MRSA, based on previously published results, estimated the prevalence of *mecC*-MRSA in the human subgroup at 0.004% (95% CI = 0.002–0.007), and the prevalence in the animal subgroup to be 0.098% (95% CI = 0.033–0.174) [75]. 

### 2.2. mecC-MRSA Human Case Reports

A total of 61 human case reports associated with *mecC*-MRSA isolates has been described (Table 3) [17,36,37,45,76,77,78,79,80,81]. Although *mecC*-positive isolates have been identified in Asia, Europe, and Oceania [21,82,83] in different hosts, all human case reports were described in European countries (Table 3). This was to be expected considering that the majority of the papers in which *mecC*-MRSA has been detected in both animals and humans, as well as in environmental samples, have been focused on countries on this continent [8,21,22,23]. 

In 4 of the 61 human case reports, *mecC*-MRSA was only identified in screen swabs (for colonization detection), with it not being related to the cause of the patient’s admission [36,37,45], and the clinical information was not indicated in another two case reports [17]. In the remaining 56 studies, *mecC*-MRSA isolates were related to (number of cases): skin and wound infections (47 cases) [37,76,79,81], joint and bone infections (3 cases) [37,77,78], respiratory infections (2 cases) [76] and bacteremia (2 cases) [37,80]. Taking into consideration the type of samples in which *mecC*-positive isolates have been detected in humans (Table 1, Table 2, Table 3 and Table 4), most *mecC* human cases were implicated in skin or wound infections. However, the detection of *mecC*-MRSA isolates in other types of samples such as blood, sputum or urine is remarkable (Table 4). Pertinently, some serious infections have been described, such as severe bone infections [78], nosocomial pneumonia [33] and bacteremia [16,24,80], in some cases ending with the death of the patient [37].

## 3. Risk Factors for *mecC*-MRSA Infection

### 3.1. Contact with Animals

Since the first description of the *mecC* gene, contact with animals has been considered to be a risk factor for *mecC-*MRSA infection or carriage for several reasons [16,17]. This gene was identified in isolates belonging to CC130, and this clonal complex was predominantly detected among methicillin-susceptible *S. aureus* (MSSA) isolates from bovine sources [17]. Moreover, the discovery of this gene in isolates obtained from dairy cows suggested that these animals might provide a reservoir of this resistance mechanism [16]. Thereby, in some of the studies carried out since then, information about the possible contact of patients with animals was indicated (Table 1 and Table 3). Many studies found out that most of the patients lived in rural areas or areas with a high density of farms [18,24,26,29,36,76,79]. In this sense, four studies indicated patient contact with livestock or farm animals [18,24,76,78,81], two referred to only contact with pets [38,45], two patients had no contact with animals and the authors did not have a plausible explanation for the detection of these isolates [37,80], one patient was a veterinarian [33], and in several studies this information was not indicated [16,17,27,30,31,41,77]. Interestingly, *mecC*-MRSA transmission between animals and humans was demonstrated in two human infection cases by whole genome sequencing. Specific clusters including isolates from each human infection case and their own livestock were detected. Thus, human and animal isolates from the same farm only differed by a small number of SNPs [18]. These findings highlight the role of livestock as a potential reservoir for *mec*C-MRSA.

### 3.2. mecC-MRSA Carriage in Humans

*S. aureus* shows a great capacity to colonize the skin and nares of hosts, being able to last over time and cause opportunistic infections [84,85]. *mecC*-MRSA isolates were identified as commensals in several prevalence and case report studies (see screen swab in Table 1, Table 2, Table 3 and Table 4). At least 54 *mecC-*MRSA positive isolates were obtained from screen swabs, mainly from the nose, but also from throat and groin sites. Moreover, isolates obtained from other types of samples could also be considered as commensals, as in one human case report in Spain in which the isolate recovered from the urine of one patient was considered as a colonizer since the patient did not present urinary symptoms [37].

*mecC*-MRSA isolates implicated in both colonization and infection were obtained from the same patient in some studies [18,37]. Indeed, one patient with bacteremia due to an *mecC*-MRSA isolate also presented nasal colonization by the same *mec*C-MRSA isolate (with the same genetic characteristics) [18]. These results corroborated the importance of colonization being the previous step, which enables isolates causing severe disease. Interestingly, in another bacteremia case in which the patient died, a household transmission between grandfather and grandson was detected, with the grandson being colonized by the same isolate [37]. Nevertheless, in other studies, *mecC-*MRSA isolates were not identified as colonizers from patients with *mecC* infections [81], and it has been suggested that *mecC*-MRSA isolates might be worse colonizers and less contagious in humans than *mecA*-MRSA isolates [76]. In the study carried out in Sweden, only two out of the patient’s 27 family members were positive for *mecC*-MRSA isolates and the median time for *mecC* carriage was 21 days [76].

### 3.3. Patient Age

Most of the patients described in case reports (Table 3) were middle-aged or elderly [17,36,37,45,77,78,79,80], except two patients: one of them was a 34 year-old farm worker with high contact with animals who presented a superficial skin lesion [81], and the other was a healthy 3 year-old child [37]. The average age of patients with *mecC*-MRSA detected in Denmark during 2007–2011 was 51 [24] and the average detected in Sweden in 2005–2014 was 60 [76]. In the Danish study, CA-MRSA *mecC* patients were significantly older than other CA-MRSA cases, indicating that *mecC*-MRSA seems to have a different origin and epidemiology to typical CA-MRSA [24]. 

### 3.4. Underlying Chronic Disease

Remarkably, in the 45 human cases detected in Sweden, most patients had some kind of underlying chronic disease (diabetes mellitus, cancer, autoimmune diseases or atherosclerotic diseases), or an existing skin lesion [76]. Infection by *mecC*-MRSA of wounds has also been suggested by others [79]. Moreover, *mecC-*MRSA infections were identified in patients with primary pathologies (diabetes, myelodysplastic syndrome, peripheral arterial occlusion disease, etc.) in one study in Austria [38], and in a patient with an urothelial carcinoma in Spain [80]. Unfortunately, information about other underlying diseases of *mecC*-MRSA positive patients is missing in most of the papers.

## 4. Characterization of *mecC*-MRSA Human Isolates

### 4.1. Clonal Lineages of mecC-MRSA of Human Origin

**Figure 1:**
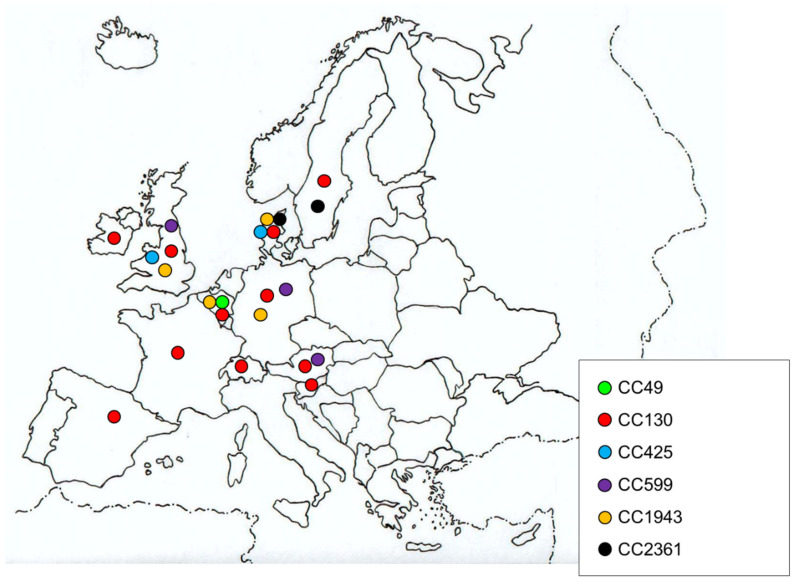
Clonal complexes (CCs) detected in *mecC*-MRSA human isolates.

As in other hosts, most of the *mecC-*MRSA isolates obtained from human samples belonged to CC130 (Table 1 and Table 3) (Figure 1). Other clonal complexes identified were CC49, CC425, CC599, CC1943 and CC2361 [16,24,25,29,30,31,38,40,41,76] (Table 1) (Figure 1). Worryingly, it has been hypothesized that SCC*mec* XI (the SCC element that contains the *mecC* gene) might have the potential to be transferred to other *S. aureus* clonal lineages due to the fact that it is bounded by integration site sequence repeats and that it has intact site specific recombination components [16] (Table S1 and S2). Until now, *mecC*-MRSA CC130 isolates have been identified in all countries in which clonal lineages were determined and it was the unique CC detected in Spain, France, Ireland, Slovenia and Switzerland [17,37,45,77,78,79,81] (Figure 1). Remarkably, in France and Spain there were several human infection reports, but in all of them the *mecC*-MRSA isolates belonged to CC130 (Table 3). After CC130, the clonal complexes CC1943 and CC599 were the most widely detected in humans, being identified in four and three countries respectively [16,25,29,31,38,41] (Figure 1). Conversely, CC49 was only described in one study in Belgium [29]. While CC49, CC130, CC425, CC599 and CC1943 were also identified in *mecC*-MRSA isolates from a non-human origin, CC2361 has been only described in humans so far [24,76]. Thus, CC130 was described in farm, domestic and wild animals and in food samples; CC49 in horses and small mammals, CC425 in wild animals and food, CC599 in pets and farm animals and CC1943 in pets [8].

**Figure 2:**
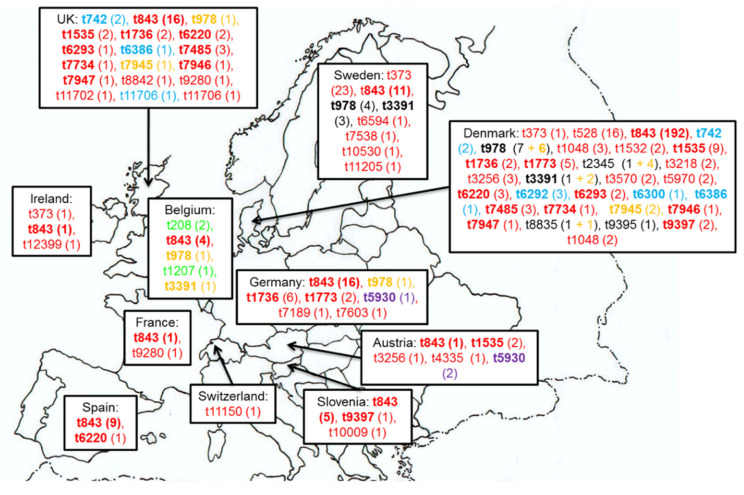
*spa* types detected in *mecC*-MRSA isolates of humans. Colors indicate the clonal complexes associated with each *spa* type: green CC49, red CC130, blue CC425, purple CC599, orange CC1943, black CC2361. The number of isolates of each *spa* type is indicated in parentheses (to calculate the number of isolates in human case reports, only one isolate from each *spa* type and each patient was considered)

A large variety of *spa* types was detected among the human *mecC*-MRSA isolates (Figure 2). The most predominant *spa* type was t843, which is associated with CC130 and was identified in a total of 260 human isolates. This *spa* type was detected in all countries in which human *mecC*-MRSA isolates were detected, except in Switzerland [45]. Other *spa* types were also described in several countries. Some of them were identified only in two countries, this is the case of t792, t1773, t5930, t6293, t6386, t7485, t7734, t7945, t7946, t7947 and t9397, but others were more widely spread as t978, t1535, t1736, t3391 or t6220 (Figure 2). Although there is a strong association among *spa* types and MLST clonal complexes [86], some *spa* types were associated with different clonal complexes. Two isolates obtained in screen swabs from two patients in two different hospitals from England presented the *spa* type t11706 [40]; one of these isolates belonged to ST1245 (CC130) and the other one to ST425 (CC425). Moreover, the *spa* types t978, t2345, t3391 and t8835 were associated in some studies with CC1943 [16,25,29], and in others with CC2361 [24,76]. Nevertheless, the founders of both clonal complexes, ST1943 and ST2361, are Single Locus Variant (SLV) of each other (and only differ at the *aroE* allele), which could explain these results (Table S1).

### 4.2. Treatment and Antimicrobial Resistance Profile of mecC-MRSA Human Isolates

Most of the human *mecC*-MRSA isolates detected were susceptible to all non-beta-lactam antimicrobials tested (Table 1 and Table 3). This is in accordance with results obtained in *mecC*-MRSA isolates from other origins [21]. In one study performed in Spain, isolates using this criterion were selected in order to identify *mecC*-MRSA or CA-MRSA isolates [69]. Although *mecC*-MRSA was not detected, this resistance phenotype was a valuable marker for Panton-Valentine Leukocidin (PVL)-producer isolates (Table S1). Nevertheless, the low prevalence of *mecC*-MRSA isolates in this region could be responsible for this result and the use of this phenotype to suspect the presence of the *mecC* mechanism should not be discarded.

The most important problem for treating *mecC*-MRSA infections is that these isolates must be correctly identified. Although *mecC* isolates are considered to be MRSA, these isolates sometimes show borderline susceptibility results for oxacillin or cefoxitin, being identified as MSSA if the *mecA* gene is only tested [44]. This could lead to the implementation of inappropriate therapies. Resistance development to other antimicrobial agents could be added to this, considering the capacity of *S. aureus* to acquire resistance to various antimicrobial agents. Some *mecC*-MRSA isolates detected in humans have shown resistance to non-beta-lactam antimicrobials [24,33,40,41,76] (Table 1). Fluoroquinolone resistance was identified in two isolates in Germany [33] and in one isolate in Denmark [24]. Macrolide and lincosamide resistance was also detected in some studies: one erythromycin resistant isolated in the UK [41] and one erythromycin and clindamycin resistant isolate in Sweden [76] and England [40]. Regarding resistance mechanisms, in two studies carried out in Ireland, the gene *sdrM*, which encodes a multidrug efflux pump related to norfloxacin resistance and *tet* efflux related to tetracycline resistance, were identified in one and two *mecC-*MRSA CC130 isolates, respectively [17,26]. Although there has only been one study, whose objective was to compare different diagnostic tests, human *mecC*-MRSA isolates resistant to several antimicrobial families have been detected [87]. The presence of resistance to non beta-lactam agents in *mecC*-MRSA isolates significantly limits our therapeutic options.

### 4.3. Virulence of mecC-MRSA Human Isolates

The search for virulence genes in human *mecC*-MRSA isolates has been highly variable from one study to another. In any case, for the moment, the most common virulence genes detected have been *hla*, *hld*, *hlb*, *edinB*, *lukED*, *cap8* or *ica*, with these genes being highly associated with CC130 [17,26,31,33,36,38,41]. Fortunately, no *mecC*-MRSA isolates carrying the PVL genes were detected. However, other clonal lineages associated with animals have been able to acquire this virulence factor [88]. For this, their detection in *mecC*-MRSA isolates cannot be ruled out in the future. Significantly, some pyrogenic toxin superantigen (PTSAg) genes have been detected in *mecC*-MRSA isolates [29,31,38,41]. These genes might be related to specific clonal lineages such as CC599, CC1943 or CC2361. Thus, the gene *tst* encoding the toxic shock syndrome toxin has been found in three CC1943 isolates (two harboring *sec* gene and one containing *seg* and *sei* genes) [29,41], in three CC599 isolates (two of them positive for *sel* gene and one for *sec* and *sel*) [31,38] and in one *sec, seg, sei, sel, sen, seo* and *seu* positive CC2361 isolate [31] (Table S1).

## 5. *mecC*-MRSA Problem: What is its Origin? Is It an Emerging Problem?

The oldest known *mecC*-MRSA isolate, dated in 1975, was detected in the retrospective study performed by García-Álvarez et al. [16] This isolate was identified in a human blood sample from Denmark and its detection suggested a possible human origin for the *mecC* gene [16]. Later, in two other retrospective studies also carried out in Denmark, two *mecC*-positive isolates were identified in samples dated in 1975 [24,25], both were also identified in human blood samples [24,25]. However, in two of these studies, the oldest sample studied was obtained in 1975, so the presence of older isolates cannot be ruled out [16,25]. With respect to the remaining retrospective studies in which the presence of the *mecC* gene was sought, the dates of the samples were much later than these three studies, with them being isolates obtained from the year 2000 and later (Table 1). Regarding the earliest reported *mecC*-MRSA isolate in other hosts, 1975 also seems to be the key date [89,90]. Therefore, this resistance mechanism might have been present for over 45 years.

Moreover, this resistance mechanism is highly associated with CC130 since most of the *mecC*-MRSA isolates belong to this clonal lineage. A human-to-bovine host-jump of CC130, which occurred ∼5429 years ago, has been suggested [91]. The time and host in which CC130 MSSA isolates acquired the *mecC* resistance gene remain unknown today. In order to establish a possible human or animal origin for the detected *mecC*-MRSA isolates in human samples, several studies analyzed the presence of IEC (immune evasion cluster) genes [17,24,26,31,33,36,41,42,78,81] (Table 1 and Table 3). In all cases, human *mecC*-MRSA isolates were negative for *sak*, *chp* and *scn* except for one ST1945 (CC130) isolate obtained from a screen swab from a patient in the UK that was positive for *sak* and *scn* (IEC type E), suggesting a possible human origin [41]. Nevertheless, it has been suggested that IEC type E might be a conserved part of ST1945 since *mecC* MRSA ST1945 isolates from wild animals also showed IEC type E in several studies in Spain [41,63,92]. 

The newest human *mecC*-MRSA isolates detected so far in Europe were obtained in 2015, one of them in Germany [27], and the other in England [42]. Both strains showed similarities to those identified in the first studies [16,17] and both belonged to CC130. Nevertheless, after phylogenetic analysis, the strain identified in England seemed not to be highly related to any of the published sequenced *mecC*-MRSA CC130 isolates [42]. Despite the non-description of *mecC*-MRSA isolates in humans in the last 5 years in Europe, data provided by previous studies have detected an increasing tendency in the prevalence of *mecC*-positive isolates [24], indicating that surveillance in detecting this resistance mechanism must be maintained. The lack of detection could be due to the low prevalence of this resistance mechanism and/or problems in *mecC* diagnostic methods. Important difficulties in the detection of *mecC*-MRSA isolates have been indicated [44,93]. It has been shown that various clinical tests used in hospital labs might have failed to identify 0 to 41% of *mecC*-MRSA isolates [93]. It is important to optimize and develop new testing protocols and redefine currently available phenotypic testing methods [44]. In this regard, several commercial PCR-based tests that detect *mecC* and *mecA* genes have been developed. Moreover, recently, *mecA*/*mecC* MRSA isolates from cattle have been described [83]. The possible clinical impact of isolates carrying both genes is currently unknown.

## 6. Implications in Veterinary and Food Safety

Although this review is focused on the human health implications of *mecC*-MRSA isolates, the effects that these isolates can have on veterinary medicine should not be forgotten. *mecC*-MRSA isolates causing infections in domestic animals have been identified in several studies [8,94,95]. However, this resistance gene seems to be most frequently detected in livestock animals including cattle, sheep and rabbits [8,21]. Although *mecC*-MRSA rarely causes clinical disease in these food-producing animals, there are reports of bovine mastitis in several countries [96,97]. As observed in humans, most of the *mecC*-MRSA isolates detected in other hosts also belong to CC130, with the characteristics of these animal *mecC*-MRSA isolates being very similar to those detected in humans [8,21].

On the other hand, the presence of *mecC*-MRSA in dairy animals is highly relevant since it could be a route of entry of these isolates into the food chain. Indeed, milk samples have been identified carrying *mecC*-MRSA [8] with the consequent risk of colonization for food handlers. In this case, it is worth highlighting one of the clinical cases included in this review in which the patient was a cheese producer [81]. *mecC*-MRSA zoonotic transmission has been demonstrated in some studies [18], with the correct prevention, detection and control measures in veterinary and food safety laboratories being necessary.

## 7. Conclusions and Future Problems Associated with *mecC*

Currently, the prevalence of human *mecC-*MRSA infections is very low. However, *mecC*-MRSA isolate transmission between different hosts indicates the great capacity of these isolates for spreading. There is a wide range of reservoirs in wild, livestock and companion animals and zoonotic transmission of these isolates could increase the number of *mecC-*MRSA human clinical cases. Moreover, SCC*mec* XI might have the potential to be transferred to other clonal lineages in the future. Their transfer to more virulent and better-adapted human clones would be deeply troubling. Worryingly, the *mecC* gene has already been detected in clonal lineages in which important virulence genes were identified (CC599, CC1943 or CC2361) or in which IEC was described (ST1945-CC130). Moreover, *mecC*-MRSA isolates resistant to non-beta lactams have been detected. Acquisition of non-beta lactam resistance by *mecC*-MRSA isolates significantly limits our therapeutic options. *mecC*-MRSA should be taken into consideration in hospital and veterinary laboratories and in food safety institutions, and prevention strategies must be implemented in order to avoid possible emerging health problems.

## Figures and Tables

**Table 1:** Human studies related to *mecC*-MRSA isolates in which prevalence can be estimated ^1^

Reference	Country	Sampling Date	Prevalence: *mecC* Positive Isolates/*S. aureus* or Methicillin Resistant *S. aureus (*MRSA) (%)	Type of Sample/Infection (Number of Isolates)	Clonal Complex: Sequence Type ^2^ (Number of Isolates)	IEC ^3^ (Number of Isolates)	Non-beta lactam Resistance (Number of Isolates) ^4^	Possible Relationship with Animals
[18,24]	Denmark	1960–2011	112 (0.21%)/53746 MRSA	Wound (37), skin (26), blood (8), post-operative wound (5), urine (4), eye/ear (2), impetigo (2), unknown (28)	CC130 (98)/CC2361 (14): ST2173, ST2174	Negative (2)	Q (NOR) (1), S (111)	Most were from rural areas (106): 4 with contact with animals
[16]	United Kingdom (UK) and Denmark	1975–2011	51 (0.04%)/approximately 120500 *S. aureus*	Screen swab (10), Skin and soft tissue infections (7), nose (5), wound (5), blood (4), skin (4), nose/mouth (2), ear (1), eye/ear (1), finger (1), fluid (1), hand (1), PEG site(1), sputum (1), toe (1), unknown (6)	CC130: ST130 (18), ST1245 (3), ST1764 (3), ST1945 (3), ST1526 (1), ST1944 (1), n/d (17)/CC1943: ST1943 (1), ST1946 (1)/CC425: ST425 (3)	-	S (51)	-
[25]	Denmark	1975–011	127/-: in routine testing 12 (5.91%)/203 MRSA	-	CC130 (107): ST130, ST1245, ST1526, ST1945/CC1943 (14): ST1943, ST1946, ST2173, ST2174/CC425 (6): ST425	-	-	-
[26]	Ireland	2000–2012	1 (1.14%)/88 MRSA	-	CC130 (1)	Negative (1)	Q (NOR) (1)	Patient lived on a Farm
[27]	Germany	2000–2016	2 (0.16%)/1277 MRSA	-	CC130 (2)	-	-	-
[28]	Austria	2002–2012	1 (0.31%)/327 MRSA	-	CC130 (1)	-	-	-
[29]	Belgium	2003–2012	9 (0.18%)/4869 *S. aureus*	Screen swab (4), urine (2), wound (2), sputum (1)	CC130 (4)/CC49 (3)/CC1943 (2)	-	S (9)	Most were from a rural area with a high density of cattle farms
[30,31]	Germany and The Netherlands	2004–2011	16/−	nasal swab (11), wound (2), joint aspirate (1), mouth swab (1), sputum (1)	CC130 (14)/CC1943: ST2361 (1)/CC599: ST599 (1)	Negative (16)	S (1)	-
[32]	Germany	2004–2005 2010-2011	2 (0.06%)/3207 MRSA	Screen swab (1), sputum (1)	-	-	-	-
[33]	Germany	2006–2011	11 (0.09%)/12691 MRSA	Wound (8), dermatitis (1), nasal swab (1), nosocomial pneumonia (1)	CC130 (11)	Negative (11)	Q [CIP (1), MFL (1)] (2), S (9)	Veterinarian (1)
[34,35]	UK	2006–2012	2/−	Screen swab (2)	CC130 (2)	-	-	-
[36]	Slovenia	2006–2013	6 (1.52%)/395 MRSA	Wound (4), Screen swab (2)	CC130: ST130 (6)	Negative (6)	S (6)	Most were from rural areas
[37]	Spain	2008–2013	2 (0.04%)/5505 *S. aureus*	Joint fluid (1), wound (1)	CC130: ST1945 (2)	-	S (2)	-.
[38]	Austria	2009-2013	6 (2%)/301 *S. aureus*	blood (2), screen swab (2), wound (2)	CC130: ST130 (3), new SLV (1)/ CC599: ST599 (2)	-	S (6)	Contact with pet rabbit (1), unknown (5)
[39]	Denmark	2010-2011	6 (6.32%)/95 MRSA	-	-	-	-	-
[40]	England	2011-2012	9 (0.45%)/2010 MRSA	Screen swab (6), wound (2), leg ulcer (1)	CC130: ST130 (2), ST1245(4), ST2573 (1), ST2574 (1)/CC425: ST425 (1)	-	L (CLI) (1) -M (ERY) (1), S (8)	-
[41]	UK	2012-2013	12 (0.53%)/2282 MRSA	Screen swab (9), SSTI (3)	CC130: ST1245 (6), ST130 (2), ST1945 (1), ST2574 (1)/CC425: ST425 (1)/CC1943: ST1943 (1)	Negative (11)/type E (1)	M (ERY) (1), S (11)	-
[42]	England	2015	1 (0.08%)/1242 MRSA	Screen swab (1)	CC130: ST130 (1)	Negative (1)	S (1)	-
[43]	England	2018-2019	1 (0.7%)/142 *S. aureus*	-	-	-	-	-
[44]	Germany, UK, Belgium	-	80/-	-	-	-	-	-

^1^ Case reports were also analyzed in some other studies but, in this table, only results from prevalence studies are included. ^2^ CC, clonal complex; ST, sequence type; ^3^ IEC, immune evasion cluster; ^4^ L, resistant to lincosamides (CLI, clindamycin); M, resistant to macrolides (ERY, erythromycin); Q, resistant to fluoroquinolones (CIP, ciprofloxacin, MFL, moxifloxacin, NOR, norfloxacin); S, susceptible to all non-beta lactam agents tested. UK, United Kingdom.

**Table 2:** Studies performed on humans, in which *mecC*-MRSA isolates were sought but not detected.

Reference	Country	Sampling Date	Type of Samples ^1^	Number of Samples or (*S. aureus* or MRSA) Isolates
[45]	Switzerland	2005–2012	Clinical/screening	1695 *S. aureus* isolates
[46]	Ghana	2007–2012	Clinical	9834 blood samples
[47]	Turkey	2007–2014	Clinical	1700 *S. aureus* isolates
[48]	Belgium	2009–2011	Screening	149 farmers and family members (41 MRSA isolates)
[49]	Hungary	2009–2011	Screening	878 children
[50]	United States	2009–2011	Clinical/screening	364 *S. aureus* isolates (102 MRSA isolates)
[51]	Ireland	2011	Screening	64 residents
[52]	UK	2011	Screening	307 cattle veterinarians
[53]	Jordan	2011–2012	Screening	716 humans (56 MRSA isolates)
[54]	Germany	2011–2013	Screening	1878 non-hospitalized adults
[55]	Belgium	2012–2013	Clinical	510 cystic fibrosis patients
[56]	Greece	2012–2013	Screening	18 veterinary personnel
[57]	The Netherlands	2012–2013	Clinical/screening	13,387 samples
[58]	UK	2012–2013	Clinical	500 *S. aureus* isolates
[59]	Egypt	2013	Clinical/screening	1300 dental patients
[60]	Taiwan	2013–2014	Clinical/screening	3717 *S. aureus* isolates
[61]	Turkey	2013–2014	Screening	7 MRSA isolates
[62]	Turkey	2013–2016	Clinical/screening	494 MRSA isolates
[63]	Spain	2014	Screening	15 humans in contact with animals
[64]	Poland	2014–2016	Screening	955 students (only one MRSA isolate)
[65]	Germany	2015	Clinical	140 Gram-positive isolates
[66]	UK	2015	Clinical	520 *S. aureus* isolates
[67]	United States	2015	Screening	479 patients
[68]	India	2015–2017	Screening	32 animal handlers
[69]	Spain	2016	Clinical/screening	45 non-beta-lactam susceptible MRSA isolates
[70]	Greece	2016–2017	Screening	68 farmers
[71]	Denmark	2017	Screening	16 workers at wildlife rehabilitation centres
[72]	Italy	2017–2018	Clinical/screening	102 MRSA isolates
[73]	Egypt	-	Screening	223 health care personnel
[74]	Madagascar	-	Screening	1548 students and healthcare workers

^1^ Screening: isolates obtained in epidemiological studies for colonization detection.

**Table 3:** Human *mecC* MRSA case reports.

Reference ^1^	Country	Sampling Date	Number of Described Case Reports	Year-Old Patient	Type of Sample/Infection ^2^	Clonal Complex: Sequence Type ^3^	IEC ^4^	Non-Beta Lactam Resistance ^5^	Possible Relationship with Animals
[76]	Sweden	2005–2014	45	Median age (range) 60 (2–86)	Wound, sputum, nasopharynx	CC130/CC2361	-	L-M (1 isolate), S (44 isolates)	Most were from a rural area: farmer (1), patients lived on farms (4)
[77]	France	2007	1	67	Fluid of lesion heel	CC130: ST1945	-	-	-
[37]	Spain	2008–2013	7	3, 50, 63, 64, 76, 80, 85	Blood, joint fluid, nasal screen swab, urine, wound	CC130: ST130, ST1945	-	S	No epidemiological data were available except for one patient who did not have any contact with animals
[78]	France	2010	1	48	Blood, ear fluid, retrosternal abscess	CC130	Negative	S	Contact with cows
[17]	Ireland	2010	2	64, 85	-	CC130: ST130, ST1764	Negative	S (but detection of *tet* efflux)	-
[45]	Switzerland	2011	1	59	Groin, nose, and throat screen swab	CC130: ST130	-	-	Contact with a cat
[79]	Spain	2012	1	46	Skin lesion swab	CC130	-	S	Patient lived in rural area with high density of livestock animals
[36]	Slovenia	2013	1	86	Nose and skin screen swabs	CC130: ST130	Negative	S	Patient lived on a farm and had contact with pigs, cats and dogs
[80]	Spain	2013	1	76	Blood	CC130: ST1945	-	S	No contact with livestock
[81]	Spain	2013–2014	1	34	Superficial skin lesion swab	CC130: ST130	Negative	S	Contact with livestock animals

^1^ Prevalence studies were also included in some papers but, in this table, only case report results are present. ^2^ Screen swab: sample for colonization detection. ^3^ CC, clonal complex; ST, sequence type; ^4^ IEC, immune evasion cluster. ^5^ L, resistant to lincosamides; M, resistant to macrolides; S, susceptible to all non-beta lactam agents tested.

**Table 4:** Type of sample/infection in which *mecC*-MRSA isolates have been identified among human patients.

Type of Sample/Infection	Number of Isolates ^2^	References
Screen swab ^1^	54	[16,29,30,31,32,33,34,36,38,40,41,42,45,76]
Skin lesion/dermatitis/impetigo wound/post-operative wound/skin and soft tissue infections	158	16,24,29,30,33,36-38,40,41,76,79,81
Blood	16	[16,24,37,38,80]
Urine	7	[24,29,37]
Nosocomial pneumonia/sputum/ Tracheal aspirate	7	[29,30,32,33,76]
Nose	5	[16]
Eye/ear	3	[16,24]
Fluid of heel/joint fluid	3	[30,37,77]
Mouth/Nose	2	[16]
Ear	1	[16]
Finger	1	[16]
Fluid	1	[16]
Hand	1	[16]
Percutaneous endoscopic gastrostomy site	1	[16]
Retrosternal abscess	1	[78]
Toe	1	[16]
Unknown	34	[16,24]

^1^ In screen swab: all samples in which was clearly indicated that they did not cause infection were included. However, in several studies it was not indicated whether samples were screen samples or if these samples were taken in infection sites. ^2^ In human case reports, only one isolate from the most representative infection sample was considered.

## Supplementary Materials

Supplementary Materials are available online at https://www.mdpi.com/2076-2607/8/10/1615/s1.
