# Supplementary material for: Human mecC-Carrying MRSA: Clinical Implications and Risk Factors
Source: Microorganisms. 2020 Oct 20;8(10):1615. doi: 10.3390/microorganisms8101615 (PMC7589452; doi:10.3390/microorganisms8101615)
Supplement: Supplementary file 1 [file microorganisms-08-01615-s001.pdf]

```
mecC      ATGAAAAAAATTTATATTAGTGTGCTAGTTCTTTTACTAATTATGATT------------    48
mecA      ATGAAAAAGATAAAAAT---TGTTCCACTTATTTTAATAGTTGTAGTTGTCGGGTTTGGT    57
          ******** **  * **   *** * * ** ***** ** ** *  **

mecC      ATAATAACTTGGTTATTCAAAGATGACGATATTGAGAAAACAATTAGTTCTATTGAAAAA    108
mecA      ATATATTTTTATGCTTCAAAAGATAAAGAAATTAATAATACTATTGATGCAATTGAAGAT    117
          ***     **     *  ****** * ** *** * ** ** ***  * * ****** *

mecC      GGAAACTATAACGAAGTATATAAAAATAGTTCAGAAAAATCTAAACTGGCATATGGAGAA    168
mecA      AAAAATTTCAAACAAGTTTATAAAGATAGCAGTTATATTTCTAAAAGCGATAATGGTGAA    177
            *** *  **  **** ****** ****     * *  ******   *   **** ***

mecC      GAAGAAATTGTAGATAGGAATAAAAAAATTTACAAAGATTTAAGTGTCAATAACTTAAAA    228
mecA      GTAGAAATGACTGAACGTCCGATAAAAATATATAATAGTTTAGGCGTTAAAGATATAAAC    237
          * ******    **  *    * ****** ** **   **** * ** **  *  ****

mecC      ATTACTAATCATGAAATTAAAAAAACTGGAAAAGATAAAAAGCAAGTTGATGTTAAATAT    288
mecA      ATTCAGGATCGTAAAATAAAAAAAGTATCTAAAAATAAAAAACGAGTAGATGCTCAATAT    297
          ***    *** * **** ******      *** ******* * *** **** * *****

mecC      AACATATATACAAAATATGGAACTATACGACGTAATACACAATTAAACTTTATTTATGAA    348
mecA      AAAATTAAAACAAACTACGGTAACATTGATCGCAACGTTCAATTTAATTTTGTTAAAGAA    357
          ** **  * ***** ** ** *  **    ** **    ***** ** *** ** * ***

mecC      GATAAGCATTGGAAATTAGATTGGAGACCAGACGTAATAGTACCTGGTTTGAAAAATGGA    408
mecA      GATGGTATGTGGAAGTTAGATTGGGATCATAGCGTCATTATTCCAGGAATGCAGAAAGAC    417
          ***      ***** *********   *    *** **  * ** **  ** * ** *

mecC      CAGAAAATTAATATAGAAACATTAAAATCAGAGCGAGGCAAAATAAAAGATAGAAATGGT    468
mecA      CAAAGCATACATATTGAAAATTTAAAATCAGAACGTGGTAAAATTTTAGACCGAAACAAT    477
          ** *  **  **** ****  *********** ** ** *****   ***  ****   *

mecC      ATAGAATTAGCTAAAACTGGAAATACATATGAAATCGGTATTGTCCCTAACAAAACACCC    528
mecA      GTGGAATTGGCCAATACAGGAACAGCATATGAGATAGGCATCGTTCCAAAGAATGTATCT    537
           * ***** ** ** ** ****   ******* ** ** ** ** ** ** **   * *

mecC      AAAGAAAAATATGATGATATTGCTCGTGACTTACAAATTGATACAAAAGCTATAACCAAT    588
mecA      AAAAAAGATTATAAAGCAATCGCTAAAGAACTAAGTATTTCTGAAGACTATATCAAACAA    597
          *** ** * *** * *  ** ***   **  **   ***  *  * *   *** *   *

mecC      AAAGTTAATCAAAAATGGGTTCAGCCAGATTCATTTGTACCAATTAAAAAGATAAATAAA    648
mecA      CAAATGGATCAAAATTGGGTACAAGATGATACCTTCGTTCCACTTAAAACCGTTAAAAAA    657
           ** *  ******* ***** **    *** * ** ** *** ******   * ** ***

mecC      CAAGATGAATATATAGACAAATTAATTAAATCATACAATTTACAAATAAACACTATAAAA    708
mecA      ATGGATGAATATTTAAGTGATTTCGCAAAAAAATTTCATCTTACAACTAATGAAACAGAA    717
             ********* **    * **    ***  **   ** *   **  **    * * **

mecC      AGCCGTGTTTATCCATTGAACGAAGCAACAGTACACCTTTTAGGTTATGTGGGTCCAATT    768
mecA      AGTCGTAACTATCCTCTAGGAAAAGCGACTTCACATCTATTAGGTTATGTTGGTCCCATT    777
          ** ***   *****  *     **** **   *** ** *********** ***** ***

mecC      AATTCTGACGAGTTAAAAAGTAAGCAATTTAGAAACTATAGCAAAAATACTGTTATTGGA    828
mecA      AACTCTGAAGAATTAAAACAAAAAGAATATAAAGGCTATAAAGATGATGCAGTTATTGGT    837
          ** ***** ** ******   **  *** ** *  *****   *  ** * ********

mecC      AAAAAAGGCTTAGAACGCCTCTATGATAAACAATTGCAAAACACTGATGGTTTTAAGGTA    888
mecA      AAAAAGGGACTCGAAAAACTTTACGATAAAAAGCTCCAACATGAAGATGGCTATCGTGTC    897
          ***** **  * ***   ** ** ****** *  * *** *    ***** * *   **

mecC      TCCATTGCAAATACTTATGACAATAAACCTTTAGACACATTATTGGAGAAAAAGGCTGAA    948
mecA      ACAATCGTTGACGATAATAGCAATACAATCGCACATACATTAATAGAGAAAAAGAAAAAA    957
           * ** *   *   * **  ***** *     * * ****** * *********    **

mecC      AACGGAAAAGATCTTCATTTAACTATAGATGCTAGAGTACAAGAAAGTATTTATAAACAT    1008
mecA      GATGGCAAAGATATTCAACTAACTATTGATGCTAAAGTTCAAAAGAGTATTTATAACAAC    1017
           * ** ****** ****  ******* ******* *** *** * ***********  *

mecC      ATGAAAAATGACGATGGATCTGGTACAGCATTACAACCAAAAACTGGAGAAATTTTAGCT    1068
mecA      ATGAAAAATGATTATGGCTCAGGTACTGCTATCCACCCTCAAACAGGTGAATTATTAGCA    1077
          ***********  **** ** ***** **  * ** **  **** ** *** * *****

mecC      TTGGTAAGTACCCCATCGTACGATGTTTATCCATTCATGAATGGATTAAGCAATAATGAC    1128
mecA      CTTGTAAGCACACCTTCATATGACGTCTATCCATTTATGTATGGCATGAGTAACGAAGAA    1137
           * ***** ** ** ** ** ** ** ******** *** ****  * ** **  * **

mecC      TACCGTAAATTAACTAACAATAAAAAAGAGCCTTTGCTCAACAAATTTCAAATCACTACA    1188
```

```
mecA      TATAATAAATTAACCGAAGATAAAAAAGAACCTCTGCTCAACAAGTTCCAGATTACAACT    1197
          **   *********  *  ********** *** ********** ** ** ** ** **

mecC      TCACCAGGTTCAACCCAAAAAATATTAACATCTATTATAGCCTTAAAAGAAAATAAACTA    1248
mecA      TCACCAGGTTCAACTCAAAAAATATTAACAGCAATGATTGGGTTAAATAACAAAACATTA    1257
          ************** *************** * ** ** *  *****  * ** * * **

mecC      GACAAAAATACTAATTTTGATATTTATGGTAAGGGTTGGCAAAAAGATGCATCATGGGGG    1308
mecA      GACGATAAAACAAGTTATAAAATCGATGGTAAAGGTTGGCAAAAAGATAAATCTTGGGGT    1317
          *** * ** ** * ** * * **  ******* ***************  *** *****

mecC      AATTATAATATCACAAGATTTAAAGTAGTAGACGGCAATATCGATTTAAAGCAAGCAATA    1368
mecA      GGTTACAACGTTACAAGATATGAAGTGGTAAATGGTAATATCGACTTAAAACAAGCAATA    1377
            *** **  * ******* * **** *** * ** ******** ***** *********

mecC      GAATCATCAGACAACATATTTTTTGCCCGCATTGCATTAGCATTAGGAGCCAAAAAATTT    1428
mecA      GAATCATCAGATAACATTTTCTTTGCTAGAGTAGCACTCGAATTAGGCAGTAAGAAATTT    1437
          *********** ***** ** *****  *  * *** * * ******    ** ******

mecC      GAGCAAGGTATGCAAGATTTGGGAATCGGTGAAAATATCCCGAGTGATTATCCCTTTTAT    1488
mecA      GAAAAAGGCATGAAAAAACTAGGTGTTGGTGAAGATATACCAAGTGATTATCCATTTTAT    1497
          **  **** *** ** *  * **  * ****** **** ** *********** ******

mecC      AAAGCACAAATCTCAAATAGTAATTTAAAAAATGAAATATTATTAGCAGATTCAGGATAT    1548
mecA      AATGCTCAAATTTCAAACAAAAATTTAGATAATGAAATATTATTAGCTGATTCAGGTTAC    1557
          ** ** ***** ***** *  ****** * ***************** ******** **

mecC      GGCCAAGGCGAGATACTAGTAAACCCTATACAAATTTTATCAATATACAGTGCTTTAGAA    1608
mecA      GGACAAGGTGAAATACTGATTAACCCAGTACAGATCCTTTCAATCTATAGCGCATTAGAA    1617
          ** ***** ** *****  * *****  **** **  * ***** ** ** ** ******

mecC      AATAACGGAAATATACAAAATCCTCATGTTTTACGTAAAACAAAATCTCAAATATGGAAA    1668
mecA      AATAATGGCAATATTAACGCACCTCACTTATTAAAAGACACGAAAAACAAAGTTTGGAAG    1677
          ***** ** *****  *    *****  * ***    * ** ***    ** * *****

mecC      AAAGATATTATACCTAAAAAAGACATAGATATATTAACTAATGGTATGGAACGTGTAGTT    1728
mecA      AAAAATATTATTTCCAAAGAAAATATCAATCTATTAACTGATGGTATGCAACAAGTCGTA    1737
          *** *******  * *** ** * **  ** ******** ******** ***  ** **

mecC      AATAAAACACATAGGGATGATATATACAAAAATTATGCCCGAATTATTGGTAAATCTGGC    1788
mecA      AATAAAACACATAAAGAAGATATTTATAGATCTTATGCAAACTTAATTGGCAAATCCGGT    1797
          *************  ** ***** ** * *  ******     * ***** ***** **

mecC      ACAGCAGAATTAAAAATGAATCAAGGGGAAACTGGAAGACAAATAGGTTGGTTTGTTTCA    1848
mecA      ACTGCAGAACTCAAAATGAAACAAGGAGAAACTGGCAGACAAATTGGGTGGTTTATATCA    1857
          ** ****** * ******** ***** ******** ******** ** ****** * ***

mecC      TATAATAAAAATAATCCTAATATGTTAATGGCGATTAATGTTAAAGACGTTCAAAATAAA    1908
mecA      TATGATAAAGATAATCCAAACATGATGATGGCTATTAATGTTAAAGATGTACAAGATAAA    1917
          *** ***** ******* ** *** * ***** ************** ** *** *****

mecC      GGGATGGCCAGCTATAATGCTACTATATCTGGAAAAGTTTATGATGATTTGTATGATAAT    1968
mecA      GGAATGGCTAGCTACAATGCCAAAATCTCAGGTAAAGTGTATGATGAGCTATATGAGAAC    1977
          ** ***** ***** ***** *  ** ** ** ***** ********  * ***** **

mecC      GGAAAAACTCAATTTGATATAGATCAGTAA    1998
mecA      GGTAATAAAAAATACGATATAGATGAATAA    2007
          ** ** *   ***  ********* * ***
```

Figure S1. Comparison of *mecC* (*mecA*$_{LGA251}$) and *mecA* sequences. The nucleotide sequence of *mecA* is from *S. aureus* USA300 (accession no. CP000730) and the nucleotide sequence of *mecC* is from *S. aureus* LGA251 (accession no. NC_017349).

Table 1. Concept related to *S. aureus* molecular typing and virulence factors.

Concept or term	Definition or explanation
Multilocus sequence typing (MLST)	Molecular typing technique that allows characterize isolates of microbial species using the DNA sequences of internal fragments of multiple housekeeping genes. This method gives a combination of several alleles that determine the sequence type (ST) of a microorganism
Clonal complex (CC)	Determined thanks to MLST technique is a cluster of sequence types (STs) in an eBURST diagram in which all STs are linked as Single Locus Variant (SLVs) to at least one other sequence type (ST)
Single Locus Variant (SLVs)	Strains that differ in only one loci
spa-typing	Molecular typing technique that implicates the sequencing of the polymorphic X region of the staphylococcal protein A gene (spa).
SCC*mec*	Genetic structure that contains the genes that confer resistance to methicillin (*mecA*/*mecC*), and that is integrated into the chromosome.
hla, hlb, hld	Hemolysin genes: these genes encode hemolysins that are virulence factors that allow the rupture of cell membranes
edinB	Epidermal cell differentiation inhibitor gene that encodes a exotoxin that specifically inhibit host protein RhoA
lukED	Leukotoxin gene that encodes a pore-forming toxin
cap8	Operon that encodes the type 8 capsular polysaccharide that is an essential factor for bacteria protection, especially when it infects a host, conferring anti-phagocytic ability
ica	Intercellular adhesion gene cluster that leads to the biosynthesis of polysaccharide intercellular adhesion (PIA) molecules, being related to biofilm formation
Pyrogenic Toxin Superantigen (PTSAg) genes	Group of exocellular toxins that share biochemical characteristics and that includes: the toxic shock syndrome toxin (TSST) and staphylococcal enterotoxins (SEA, SEB, SEC, SED, SEE, SEG, SEH, SEI, SEJ, SEK, SEL, SEM, SEN, SEO, SEP, SEQ, SER, SEU).
tst	Gene that encodes the toxic shock syndrome toxin (TSST) associated with severe clinical symptoms
sea, seb, sec, sed, see, seg, seh, sei, sej, sek, sel, sem, sen, seo, sep, seq, ser, seq, seu	Staphylococcal enterotoxin genes related to food poisoning
PVL (Panton-Valentine Leukocidin)	Leukotoxin related to alterations of cell permeability and leukocyte destruction

Table S2. SCC*mec* types described in *S. aureus* as combination of the *mec* complex and the *ccr* type, indicating in parentheses the structure of the *mec* complex and the *ccr* genes that constitute the *ccr* type.

**SCC*mec* type**	***mec* complex**	***ccr* type**
I	B (IS1272-Δ*mecR1*-*mecA*-IS431)	1 (*ccrA1ccrB1*)
II	A (*mecI*-*mecR1*-*mecA*-IS431)	2 (*ccrA2ccrB2*)
III	A (*mecI*-*mecR1*-*mecA*-IS431)	3 (*ccrA3ccrB3*)
IV	B (IS1272-Δ*mecR1*-*mecA*-IS431)	2 (*ccrA2ccrB2*)
V	C2 (IS431-Δ*mecR1*-*mecA*-IS431) (IS431s in opposite direction)	5 (*ccrC1*)
VI	B (IS1272-Δ*mecR1*-*mecA*-IS431)	4 (*ccrA4ccrB4*)
VII	C1 (IS431-Δ*mecR1*-*mecA*-IS431)	5 (*ccrC1*)
VIII	A (*mecI*-*mecR1*-*mecA*-IS431)	4 (*ccrA4ccrB4*)
IX	C2 (IS431-Δ*mecR1*-*mecA*-IS431) (IS431s in opposite direction)	1 (*ccrA1ccrB1*)
X	C1 (IS431-Δ*mecR1*-*mecA*-IS431)	7 (*ccrA1ccrB6*)
XI	E (*blaZ*-*mecC*-*mecR1*-*mecI*)	8 (*ccrA1ccrB3*)
XII	C2-like (ΔIS431-*mecA* -Δ*mecR1* -IS431)	9 (*ccrC2*)
XIII	A (IS431-*mecI*-*mecR1*-*mecA*-IS431)	9 (*ccr*C2-new)
XIV	A (*mecI*-*mecR1*-*mecA*-IS431)	1 (*ccrA1ccrB1*)
